# A Tribute to Marcy Carlson Speer, 1959–2007

**DOI:** 10.1371/journal.pgen.0030230

**Published:** 2007-12-28

**Authors:** Suzanne M Leal

**Affiliations:** Suzanne M. Leal (sleal@bcm.edu) Baylor College of Medicine Houston, Texas, United States of America

It is with great sadness that we say goodbye to Marcy Carlson Speer, who died on August 4, 2007, at the age of 47 after a two-year battle with breast cancer. Marcy was an extremely accomplished scientist who, at the time of her death, was the director of the Duke Center for Human Genetics and chief of the Division of Medical Genetics. During her career, she published 124 articles and 16 book chapters; the topics of her scholarly scientific work ranged from gene mapping and identification to method development in genetic epidemiology and authoritative book chapters on linkage analysis. Over her scientific career, Marcy was the recipient of 24 National Institutes of Health (NIH) grants; of these, she was principal investigator of 18.

Marcy was born on October 1, 1959, in Indianapolis and was raised in Indiana and Illinois. She graduated from Champaign Centennial High School, where she was first introduced to population genetics and became interested in both the biological and mathematical aspects of genetics. After receiving a Bachelor of Arts degree in Mathematics from Indiana University in 1981, Marcy continued her studies at Sarah Lawrence College, where, in 1983, she received a Master of Science degree in Human Genetics specializing in genetic counseling. At Duke University in Durham, North Carolina, Marcy completed her PhD in Zoology with a concentration in statistical human genetics under the mentorship of Margaret Pericak-Vance. In 1991 she moved to New York City to continue her training in statistical genetics as a post-doctoral fellow at Columbia University with Jurg Ott as her advisor. She then returned to Duke University Medical Center (DUMC) and rose to the rank of fully tenured professor, making her one of only ten female faculty members at DUMC in the Department of Medicine ever to be promoted to this position. She also held appointments in the Department of Biostatistics and Bioinformatics and the Department of Molecular Genetics and Microbiology. In 2006, she became the director of the Center for Human Genetics and chief of the Division of Medical Genetics, in the Department of Medicine, a position she held until her death.

In addition to her training in genetic epidemiology, statistical genetics, and genetic counseling, Marcy was a board-certified medical geneticist and genetic counselor. Her unique training gave her the ability to tackle a wide variety of scientific issues. One of her main interests was the genetic etiology of neurological disorders, with a particular interest in neural tube defects and Chiari type I malformations. Marcy had a long history of studying a variety of muscular dystrophies, including limb-girdle and facioscapulohumeral muscular dystrophy. She was involved in the identification of genes for hereditary hemorrhagic telangiectasia, type 2, bethlem myopathy, limb girdle muscular dystrophy 1A, and dominant intermediate Charcot-Marie-Tooth disease. She was also interested in elucidating phenotype–genotype correlations for both Mendelian and complex traits and in identifying endophenotypes for bipolar disorder. Marcy was fascinated by how genes and environment interact, which is exemplified by her work on neural tube defects, folate pathway genes, and preconception dietary folic acid supplementation. In all, Marcy did research on 24 disease phenotypes, which, in addition to those previously mentioned, included cystic fibrosis, rickets, tuberous sclerosis, pulmonary fibrosis, asthma, and sensorineural hearing loss. In the clinical arena, Marcy was actively involved in genetic counseling for neuromuscular and complex genetic diseases. Her expertise in disease etiology was recognized internationally, and she was a member of the advisory board of the American Syringomyelia Alliance Project, the chair of the American Syringomyelia Alliance Project Research Committee, and a member of the Professional Advisory Committee of the Spina Bifida Association of America. Marcy was sought out as a speaker in her areas of expertise, and was to be an invited speaker at the Neural Tube Defects International Conference in Genoa, Italy in October 2007.

In addition to her research related to disease phenotypes, Marcy also tackled issues in statistical genetics. Her work included demonstrating how neural networks can be used to aid in the determination of disease status, developing chromosome-based simulation programs for rapid generation of genotype data, and studying inflation of type I error in the presence of intermarker linkage disequilibrium. Her latest published methodological work was on the development of an extension of the likelihood ratio test, which makes use of unaffected sibling genotype data when there is missing parental data for offspring genotype risks, maternal effects, and parent-of-origin effects.

Marcy wrote eight erudite book chapters on methods in gene mapping and microarray analysis. These book chapters contain detailed information on the methodology used for linkage analysis, sample size estimation, and power calculations. These chapters are exceedingly informative to researchers who require specific knowledge on how to carry out gene-mapping studies.

Marcy was devoted to training scientists in the field of statistical genetics and genetic epidemiology. She was the director of admissions and of graduate studies for the Duke University Program in Genetics and Genomics. In addition to being a mentor to PhD students, Marcy was involved in many educational activities at Duke University and the University of North Carolina at Chapel Hill and Greensboro, which included directing and teaching courses on human, mammalian, statistical, and population genetics. Marcy was course director and instructor for an annual course, *Genetic Analysis Methods for Medical Researchers*, that was attended by scientists from around the world. This course is supported by a grant from the NIH–National Human Genome Research Institute (NHGRI), for which Marcy was the principal investigator.

On October 3, 2007, Marcy was posthumously awarded the *Marcy Speer Outstanding CSR Reviewer Award*, which was created in her honor. With this award, the Center for Scientific Review (CSR) recognized and paid tribute to Marcy's tremendous service to the NIH peer-review system. In the past 10 years, Marcy participated in over 50 study sections. Her service at the time of her death included being a permanent member of the Genetics of Health and Disease study section (formerly Mammalian Genetics) and also serving as an ad hoc member for the NIH-NHGRI and National Institute of Environmental Health Sciences (NIEHS). This spring she agreed to serve a four-year term on the NIEHS' Environmental Health Sciences Review Committee. The Marcy Speer Outstanding CSR Reviewer Award will be given annually to an individual who, like Marcy, is highly dedicated to the NIH peer-review system.

Marcy was committed not only to gaining and spreading scientific knowledge; her compassion also drove her to make advances which would improve health and quality of life. It was her strong desire to advance the translation of research findings into clinical care. She was especially dedicated to research that would aid infants and children who are afflicted with genetic diseases. For her study on neural tube defects, Marcy visited rural areas of Guatemala where she was touched by the beautiful children living in extreme poverty. It was her hope to help reduce the high incidence of neural tube defects in Guatemala and Central America, which is believed to be partially due to the joint effects of genetic susceptibility and exposure to fumonisin, a mycotoxin that often contaminates corn.

**Image 1:**
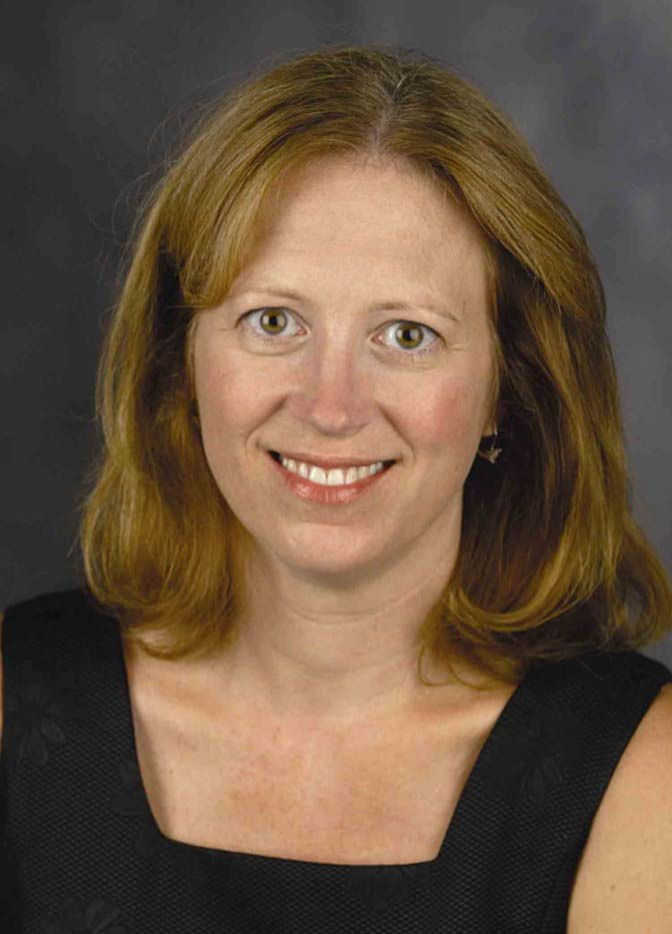
Marcy Carlson Speer

Although Marcy was an extremely brilliant and accomplished scientist, her family was the most important aspect of her life. She was devoted to her young daughters, Kira (13 years) and Casey (11 years), and to her husband, Kevin. She knew the importance of being present for both the big and little events of her children's lives, and never let her busy work schedule prevent her from attending every sporting event and ice-skating practice. Even when Marcy was sick and her boundless energy was sapped from battling breast cancer, she always had time for her family, friends, and colleagues. I was constantly amazed at Marcy's positive attitude (Image 1). She was always extremely enthusiastic about every aspect of life, from new scientific projects, to Kira and Casey's various activities. In my 16 years of knowing Marcy, I cannot recall her having a bad word to say about anyone. Even with a grim diagnosis of metastatic breast cancer, Marcy did not waste any time feeling sorry for herself; instead, she was determined not to let the disease conquer her and to live her life to its fullest, both personally and professionally. Marcy was resolved to move the Center for Human Genetics, which she headed, forward into the 21st century, and to find novel ways to elucidate the complex interplay between the environment and genetics. She was actively pursuing the specifics of how to handle this challenging problem up to the time of her death.

Marcy was enlightened in her understanding that people always come first and that being an outstanding scientist does not preclude one from being a warm, caring, and insightful person. I consider myself extremely lucky to have known this incredible lady; I will always value Marcy's friendship and her extreme kindness. She was a dear friend and the nicest person I have ever known.

Even though Marcy's life was too short, some comfort can be found in that she lived her life to the fullest and crammed more living into her 47 years than most people do who live to an advanced age. At her funeral, Marcy's brother reflected that the one word that best described her was *beautiful*. Everyone whose life was touched by Marcy would have to agree; she was, in every way, beautiful

